# Global research status analysis of the association between aortic aneurysm and inflammation: a bibliometric analysis from 1999 to 2023

**DOI:** 10.3389/fcvm.2023.1260935

**Published:** 2023-12-04

**Authors:** Qiuguo Wang, Guihuan Chen, Zhen Qi, Yifan Zeng, Ling Tan, Hao Tang

**Affiliations:** ^1^ Department of Cardiovascular Surgery, The Second Xiangya Hospital, Central South University, Changsha, China; ^2^ Department of Anesthesiology, Reproductive and Genetic Hospital of Citic-Xiangya, Changsha, China

**Keywords:** aortic aneurysm, inflammation, bibliometric analysis, frontier research hotspots, CiteSpace, VOSviewer

## Abstract

**Background:**

Aortic aneurysm is a chronic arterial disease that can lead to aortic rupture, causing severe complications and life-threatening risks for patients, and it is one of the common causes of death among the elderly. Increasing evidence suggests that inflammation plays an important role in the progression of aortic aneurysm. However, there is a lack of literature-based quantitative analysis in this field.

**Methods:**

Up to March 30, 2023, we collected 3,993 articles related to aortic aneurysm and inflammation from the Web of Science Core Collection (WoSCC) database for bibliometric analysis. The collected literature data were subjected to visual analysis of regional distribution, institutions, authors, keywords, and other information using tools such as CiteSpace, VOSviewer, the R package “bibliometric,” and online platforms.

**Results:**

The number of publications in this research field has been steadily increasing each year, with the United States and China being the main contributing countries. Harvard University in the United States emerged as the most active and influential research institution in this field. Jonathan Golledge and Peter Libby were identified as the authors with the highest publication output and academic impact, respectively. Researchers in this field tend to publish their findings in influential journals such as the Journal of Vascular Surgery and Arteriosclerosis Thrombosis and Vascular Biology. “Abdominal aortic aneurysm,” “giant cell arteritis,” “arterial stiffness,” and “smooth muscle cells” were identified as the hottest topics in the field of aortic aneurysm and inflammation. In terms of keyword co-occurrence analysis, “Clinical relevant studies of AA“ (red), “Inflammatory activation” (green), “Inflammatory mechanisms related to pathogenesis” (dark blue), “Cytokines” (yellow), “Risk factors” (purple), and “Pathological changes in vascular wall” (cyan) formed the major research framework in this field. “Inflammation-related pathogenesis” and “inflammation activation” have emerged as recent hot research directions, with “monocytes,” “progression,” and “proliferation” being the prominent topics.

**Conclusion:**

This study provides a comprehensive analysis of the knowledge network framework and research hotspots in the field of aortic aneurysm and inflammation through a literature-based quantitative approach. It offers valuable insights to guide scholars in identifying meaningful research directions in this field.

## 1. Introduction

The aortic aneurysm (AA) refers to the abnormal dilation of the arterial wall caused by structural abnormalities or damage, characterized by an expansion exceeding 50% of the original diameter of the artery. It commonly occurs in the abdominal and thoracic aorta. AA is a vascular disease of the aorta with insidious onset, lack of specific symptoms, and signs. As AA progresses, it may lead to aortic rupture or formation of an aortic dissection, resulting in high mortality and morbidity rates. Age (>60 years), hypertension, smoking, and multifactorial degenerative changes in the aorta are known important risk factors contributing to the development of AA (1). With the global ageing population and increased prevalence of hypertension, the incidence and detection rate of AA are gradually rising. Therefore, investigating the mechanisms underlying the development of AA is of great significance. Smooth muscle cell (SMC) loss, extracellular matrix degradation, and endothelial cell injury are considered the major pathological features and key factors in the development of AA. The activation of inflammatory cells and associated inflammatory mechanisms are believed to contribute to the aforementioned pathological changes and drive the progression of AA (2–4). An increasing number of studies are focusing on the role and mechanisms of inflammatory cells and inflammatory responses in the pathogenesis of AA, aiming to explore novel therapeutic approaches for AA.

Bibliometrics is an interdisciplinary science that utilizes mathematical and statistical methods to quantitatively analyze the interrelationships among various knowledge sources. It has been widely applied in analyzing research characteristics and trends, playing a crucial role in scientific research. Through the analytical methods of bibliometrics, we can gain a more comprehensive and clear understanding of the current status of a research field. Despite significant advances in the pathology and etiology research of AA, our understanding of the inflammatory cells and related inflammatory responses contributing to the onset of arterial aneurysm remains limited, lacking systematic and specialized analysis. Therefore, we employed common visualization analysis tools in bibliometrics, such as CiteSpace, VOSviewer, the “bibliometric” package in R, as well as online platform (https://bibliometric.com/app) to conduct a visual analysis of relevant literature on AA and inflammation from 1999 to 2023. Through analyzing the research characteristics and identifying the hot trends in this field, we aim to provide valuable insights for future studies.

## 2. Materials and methods

### 2.1. Data source and collation

**Figure 1:**
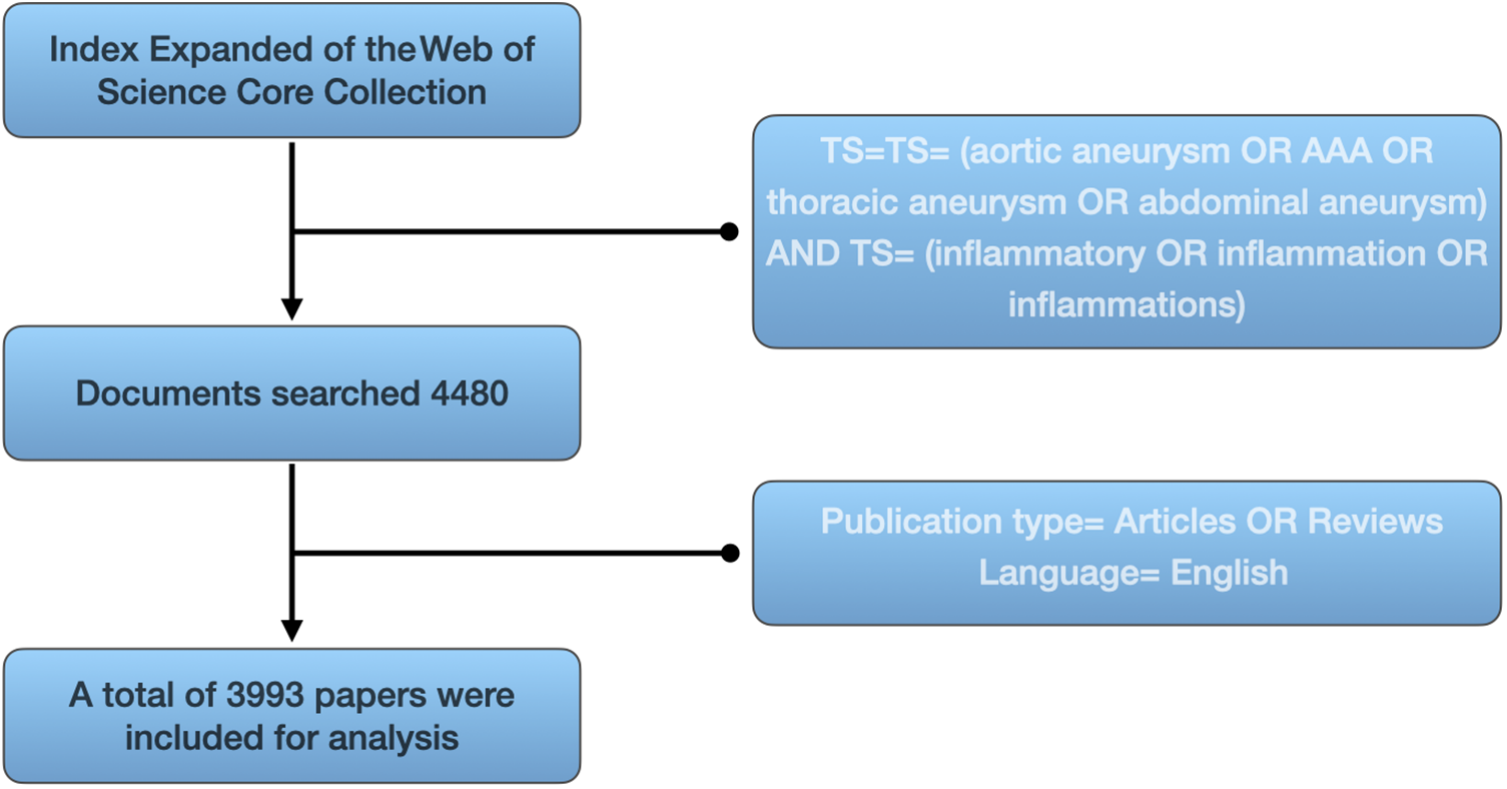
The Literatures screening flowchart.

As of March 30, 2023, we conducted a literature search in the Science Citation Index Expanded (SCI-E) of the Web of Science Core Collection (WoSCC) database. The search query used was TS = (aortic aneurysm OR AAA OR thoracic aneurysm OR abdominal aneurysm) and TS = (inflammatory OR inflammation OR inflammations). We only included literature data that met the criteria of being original articles and reviews, written in English. In total, 3,993 articles were collected (Figure 1). The literature full records, along with their cited references, were exported and organized in plain text format. The organized literature data was subjected to visual analysis using tools such as CiteSpace, VOSviewer, the “bibliometric” package in R, and online platforms.

### 2.2. Bibliometric analysis and visualization

CiteSpace and VOSviewer are powerful and commonly used bibliometric tools that facilitate the analysis and visualization of academic literature (5, 6). They enable us to gain a deeper understanding of the knowledge structure and research trends in the academic field, as well as discover new research directions and opportunities. We utilized CiteSpace (version 6.2.R4) to conduct keyword clustering analysis and explore evolutionary paths in the literature, including the dual-map overlay visualization and citation bursts of publications. Additionally, we used VOSviewer [Version 1.6.18(0)] to analyze the literature for the country and inter-institutional collaboration networks, to build networks of cited authors and co-cited authors and co-cited journals, and to analyze keywords for co-occurrence. we utilized the R package “bibliometric” to analyze the international collaborations and to analyze the influence of authors. Furthermore, we organized the literature data and imported it into the online platform (https://bibliometric.com/app) to generate a world map visualization of the literature. We also imported the data on the number of publications in different years into Microsoft Office Excel [version 16.73(23051401)] to create fitted curves representing the publication trends over time.

## 3. Results

### 3.1. Annual trend of publications

**Figure 2:**
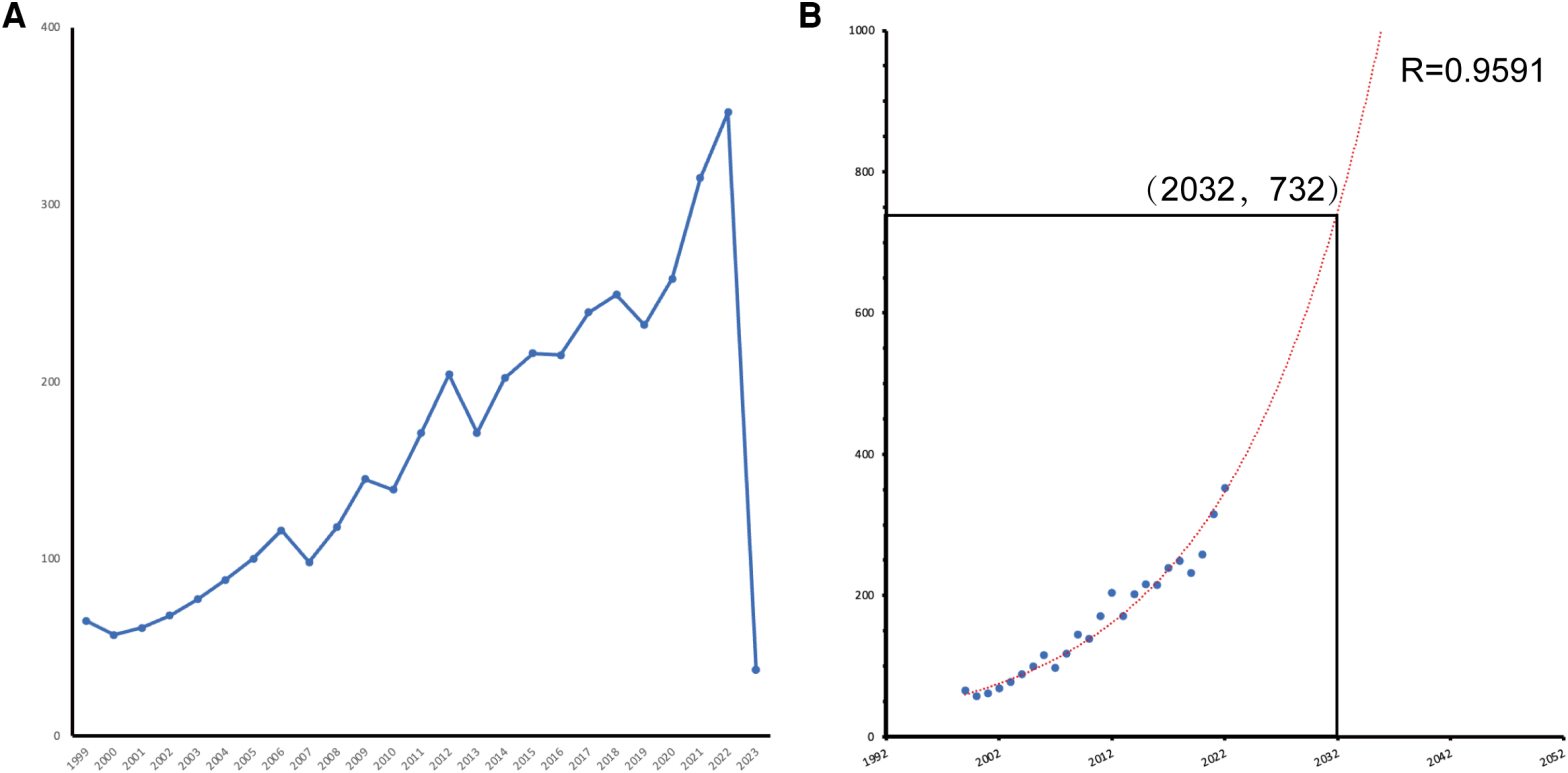
(**A**) Line diagram of the number of annual publications in the field. (**B**) Trend forecast of annual output volume (*R*² = 0.9591) with an anticipated 732 publications in 2032.

We conducted a search in the WoSCC database for research literature related to aortic aneurysm and inflammation and identified a total of 3,993 original articles and reviews published between 1999 and 2023. From 1999 to 2019, the number of publications showed a steady growth trend. In contrast, the growth in the number of papers accelerated significantly from 2019 onwards, with the peak year being 2022 (*n* = 354, 8.86%). As of 2022, the average annual publication rate was 164.96 papers (Figure 2A). Based on the fitted curve (*R*^2^ = 0.9591), we predict that in the next decade (2032), the number of publications in the field could reach 732 papers (Figure 2B).

### 3.2. Countries/regions and institutions analysis

**Figure 3:**
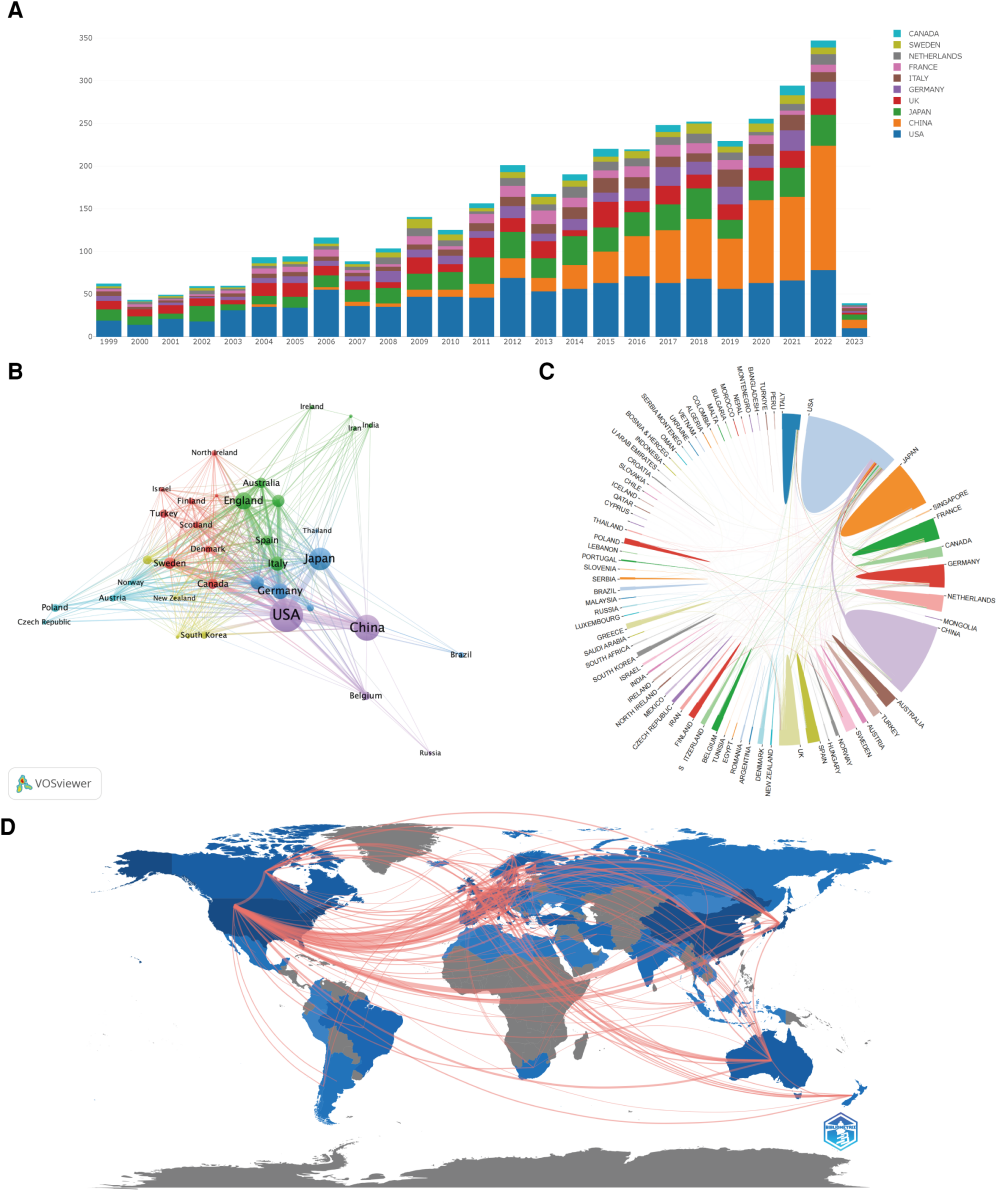
(**A**) Bar chart of the top 10 countries in annual research paper output. (**B**) Co-occurrence analysis of countries with research paper output exceeding 10. (**C**) Chord diagram of international collaborations among countries. (**D**) World geographic network map of research in this field.

**Figure 4:**
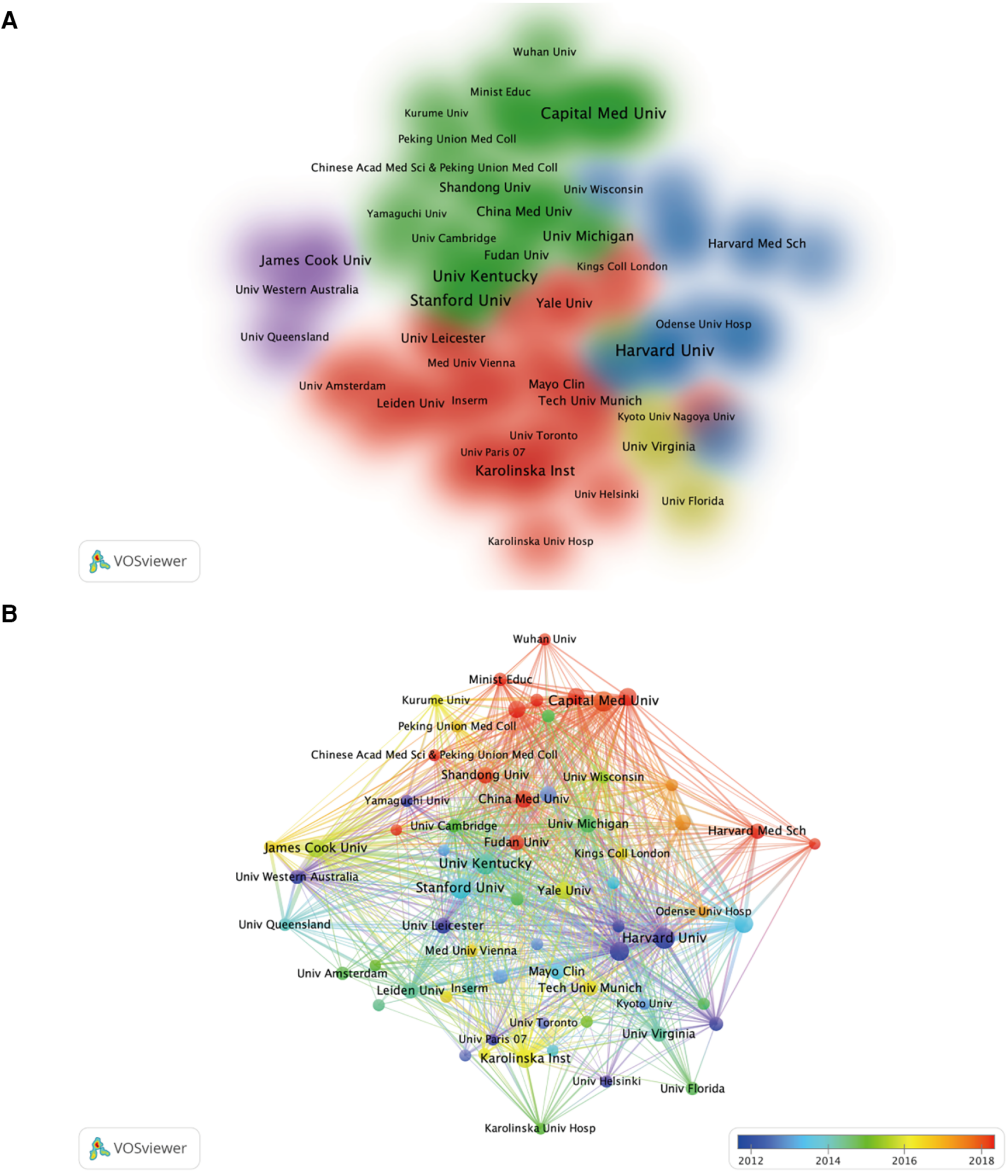
Visualization analysis of institutional collaborations in the field of AA research related to inflammation (**A**), as well as visual analysis of research institutions’ publications and collaborations in different time periods (**B**).

In the field, a total of 76 countries and regions have published 3,993 papers between 1999 and 2023 (Figure 3A). Among them, the United States (1,163 papers, 29.13%), China (711 papers, 17.81%), Japan (527 papers, 13.20%), the United Kingdom (319 papers, 7.99%), and Germany (263 papers, 6.59%) are the top five countries in terms of publication volume. They are also the top five countries with the highest citation counts (Table 1). Through co-occurrence analysis of the countries, it was found that the United States and China serve as the research cores in this field, with Japan, the United Kingdom, and Germany forming research cores of their own. There is close collaboration between high-publishing countries and other nations, particularly among the United States, China, Japan, and the United Kingdom (Figures 3B–D). By analyzing the literature, we found that the top ten institutions with the highest publication volume are primarily from the United States and China (Table 2). Among them, Harvard University, Peking Union Medical College, and Stanford University are the top three institutions in terms of publication output. It is noteworthy that China has experienced rapid growth in research output since 2016 and has become the leading country in terms of publication volume in recent years. Chinese universities and research institutions have gradually become popular research entities in this field (Figures 4A,B).

**Table 1:** Top 10 countries in number of publications and citations in the field of AA research related to inflammation.

Rank	Country	Publications	Citations
1	USA	1163	61567
2	China	711	11711
3	Japan	526	15743
4	England	319	15151
5	Germany	263	9121
6	Italy	219	6769
7	France	187	8354
8	Netherlands	156	6206
9	Sweden	142	4944
10	Canada	118	4569

**Table 2:** Top 10 institutions in numbers of papers and citations in the field.

Rank	Institution	Publications	Rank	Institution	Citations
1	Harvard Univ	69	1	Harvard Univ	7271
2	Capital Med Univ	66	2	Washington Univ	4872
3	Univ Kentucky	63	3	Univ Kentucky	3641
4	Stanford Univ	63	4	Brigham & Womens Hosp	3518
5	Washington Univ	58	5	Univ Penn	3493
6	Brigham & Womens Hosp	54	6	Stanford Univ	2704
7	Karolinska Inst	52	7	Univ Calif San Francisco	2653
8	James Cook Univ	48	8	Univ Michigan	2232
9	Huazhong Univ Sci & Technol	48	9	Kings Coll London	2209
10	Univ Michigan	47	10	Univ Western Australia	1778

### 3.3. Co-authorship and co-citation authors

**Figure 5:**
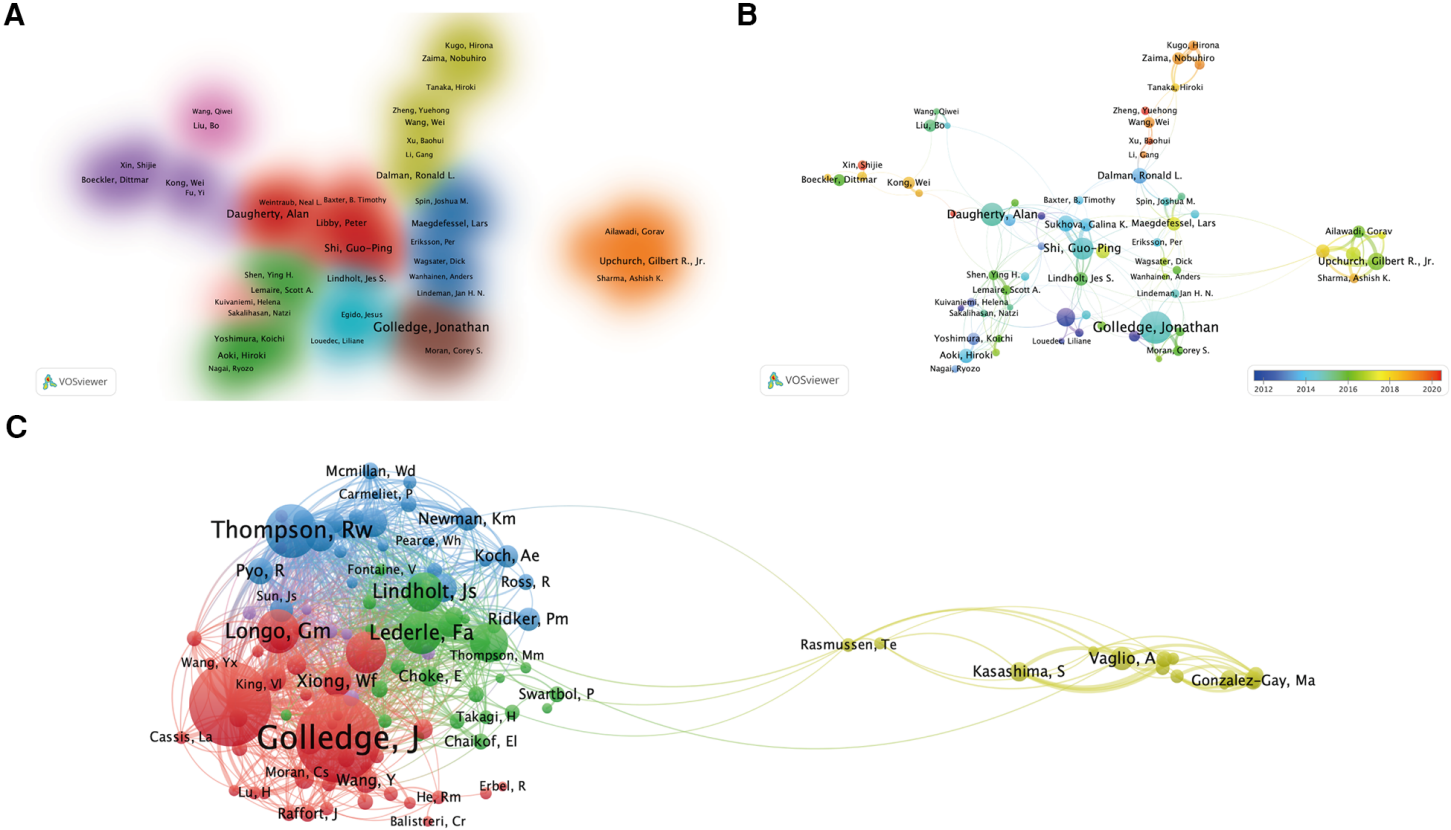
Visualization analysis of author collaborations in the field (**A**), publication volume and collaborations in different time periods (**B**), and visualization analysis of co-cited authors (**C**).

A total of 18,161 authors were included in the literature on AA and inflammation research. Approximately 4.23% of the authors had completed five or more papers. Using the “bibliometric” package in R, we conducted an analysis of the top 10 authors with the highest productivity and citation counts, as shown in Table 3. The majority of highly productive and influential scholars were from the United States and China. Jonathan Golledge, an Australian scholar, had the highest number of published papers, followed by Alan Daugherty and Gilbert R. Upchurch Jr., both from the United States. In terms of academic impact in the field, the top three authors were Peter Libby, Alan Daugherty, and Robert W. Thompson, all from the United States. Figure 5A illustrates the collaborative relationships among authors who have published ten or more papers. The main contributors to research achievements in this field were Peter Libby, Alan Daugherty, and Guoping Shi, forming a core scientific collaboration group. Additionally, they had collaborative relationships with Jonathan Golledge and Gilbert R. Upchurch Jr., who formed their own collaboration group. Furthermore, we identified the emergence of new collaborative groups after 2018, primarily represented by scholars Wei Wang and Nobuhiro Zaima from China and Japan (Figure 5B), respectively. In the author co-citation analysis (Figure 5C), it was observed that Jonathan Golledge and Alan Daugherty had similar research trends and formed the most influential research cluster in this field.

**Table 3:** Top 10 authors in the number of publications and total co-citations in the field.

Rank	High published author	Publications	Rank	High co-cited author	Citations
1	Jonathan Golledge	51	1	Peter Libby	2420
2	Alan Daugherty	36	2	Jonathan Golledge	2100
3	Guo-Ping Shi	34	3	Alan Daugherty	1865
4	Jean-Baptiste Michel	29	4	Guo-Ping Shi	1764
5	Gilbert R. Upchurch Jr	27	5	Ronald L. Dalman	1467
6	Peter Libby	25	6	Galina K. Sukhova	1457
7	Ronald L. Dalman	24	7	Jean-Baptiste Michel	1334
8	Gang Su	23	8	B. Timothy Baxter	1058
9	Hiroki Aoki	22	9	Dianna M. Milewicz	1003
10	Jie Du	21	10	Lisa A. Cassis	975

### 3.4. Analysis of journals and research domains

**Figure 6:**
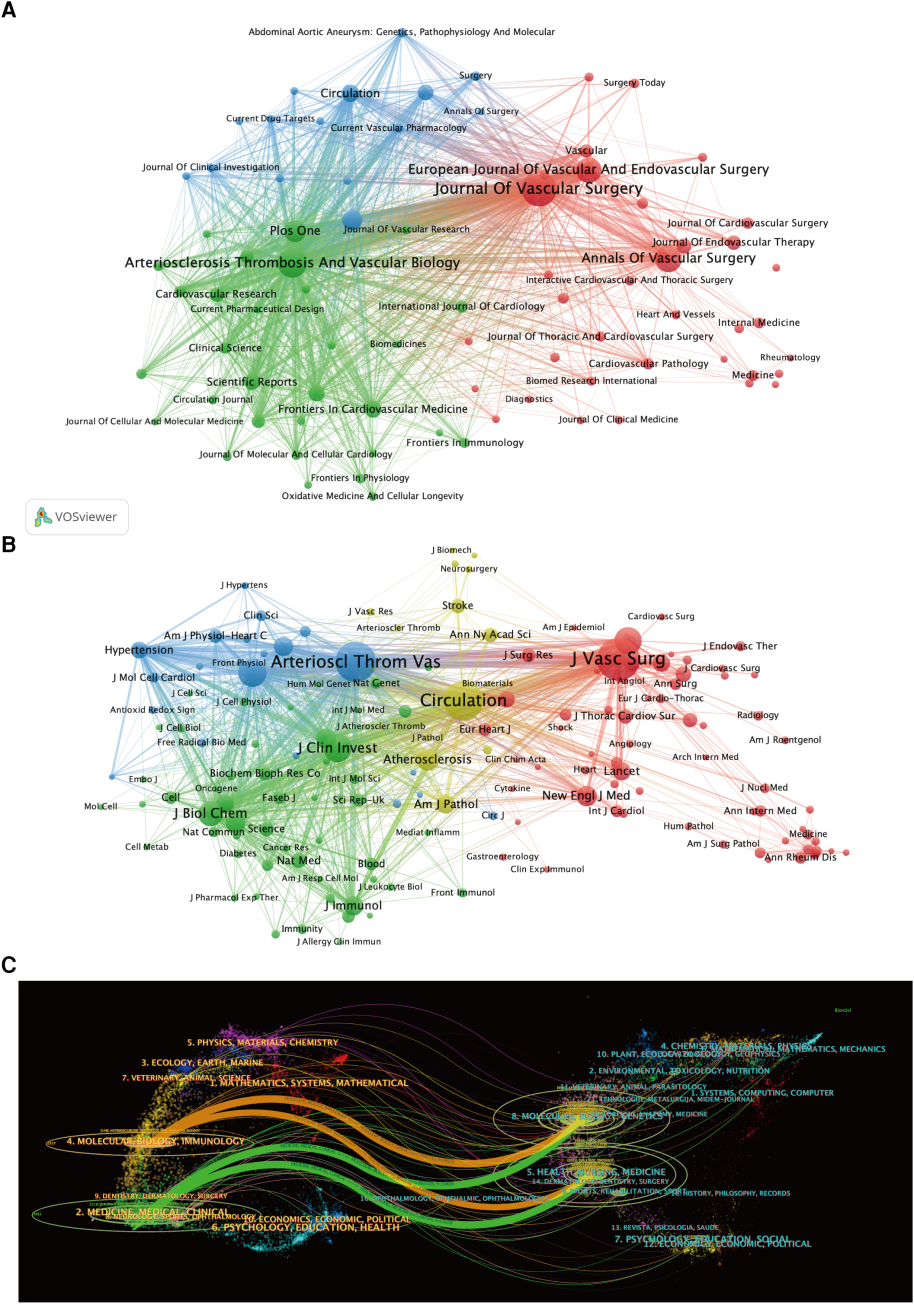
(**A**) Visualization analysis of journals with publication volume ≥10 articles. (**B**) Visualization analysis of journals with co-citations ≥200 times, and (**C**) dual-map overlay of journals in this research field.

According to the statistics, a total of 3,993 literature documents in this field were included in the study, which were published in 974 different journals. We conducted a visualization analysis of the journals in this field (with publication of 10 or more papers) using VOSviewer software (Figure 6A). The top 10 journals with the highest publication output in this field are currently the Journal of Vascular Surgery, Arteriosclerosis Thrombosis and Vascular Biology, Annals of Vascular Surgery, and European Journal of Vascular and Endovascular Surgery, all of which have published over 100 papers (Table 4). Co-cited journals refer to a group of journals that are simultaneously cited by other articles. The visualization analysis software constructs a network of journal nodes based on the citation relationships among the literature, displaying the influence and importance of these journals in the relevant field. The Journal of Vascular Surgery, Arteriosclerosis Thrombosis and Vascular Biology, Circulation, and Journal of Clinical Investigation are the most influential core journals in this field (Figure 6B; Table 5).

**Table 4:** Top 10 journals in the number of publications in the field.

Rank	Journal	Publications	IF	JCR
1	Journal of Vascular Surgery	210	4.86	Q2
2	Arteriosclerosis Thrombosis and Vascular Biology	148	10.514	Q1
3	Annals of Vascular Surgery	122	1.607	Q4
4	European Journal of Vascular and Endovascular Surgery	111	6.427	Q1
5	Plos One	81	3.752	Q3
6	Atherosclerosis	70	6.847	Q2
7	Circulation	54	39.918	Q1
8	Frontiers in Cardiovascular Medicine	50	5.846	Q3
9	Scientific Reports	47	4.996	Q3
10	International Journal of Molecular Sciences	46	6.208	Q2

**Table 5:** Top 10 journals in the number of co-citations in the field.

Rank	Journal	Citations	IF	JCR
1	Journal of Vascular Surgery	9853	4.86	Q2
2	Arteriosclerosis Thrombosis and Vascular Biology	8940	10.514	Q1
3	Circulation	8671	39.918	Q1
4	Journal of Clinical Investigation	4402	19.456	Q1
5	Circulation Research	4299	23.213	Q1
6	European Journal of Vascular and Endovascular Surgery	3933	6.427	Q1
7	Journal of Biological Chemistry	3361	5.486	Q2
8	Atherosclerosis	2650	6.847	Q2
9	New England Journal of Medicine	2295	176.079	Q1
10	Plos One	2238	3.752	Q3

The dual-map overlay of journals provides valuable insights for researchers to gain a deeper understanding of citation and collaboration relationships among different journals in the academic field, revealing the structure and dynamics of the academic network (5). Figure 6C depicts the overlay of journals based on the literature data in this study, with the left and right sides representing citing and cited journals, respectively. The colored lines connecting from citing to cited journals represent citation links, indicating the citation relationships among the journals. It is noteworthy that the elliptical shape of the journal labels vertically expands with the increasing number of published papers, while the horizontal axis widens with a greater number of authors. Figure 6C highlights four main citation pathways in orange and green. The literature published in the fields of Molecular/Biology/Immunology and Medicine/Medical/Clinical is cited by literature published in the fields of Molecular/Biology/Genetics and Health/Nursing/Medicine.

### 3.5. Citation and co-citation analysis

**Figure 7:**
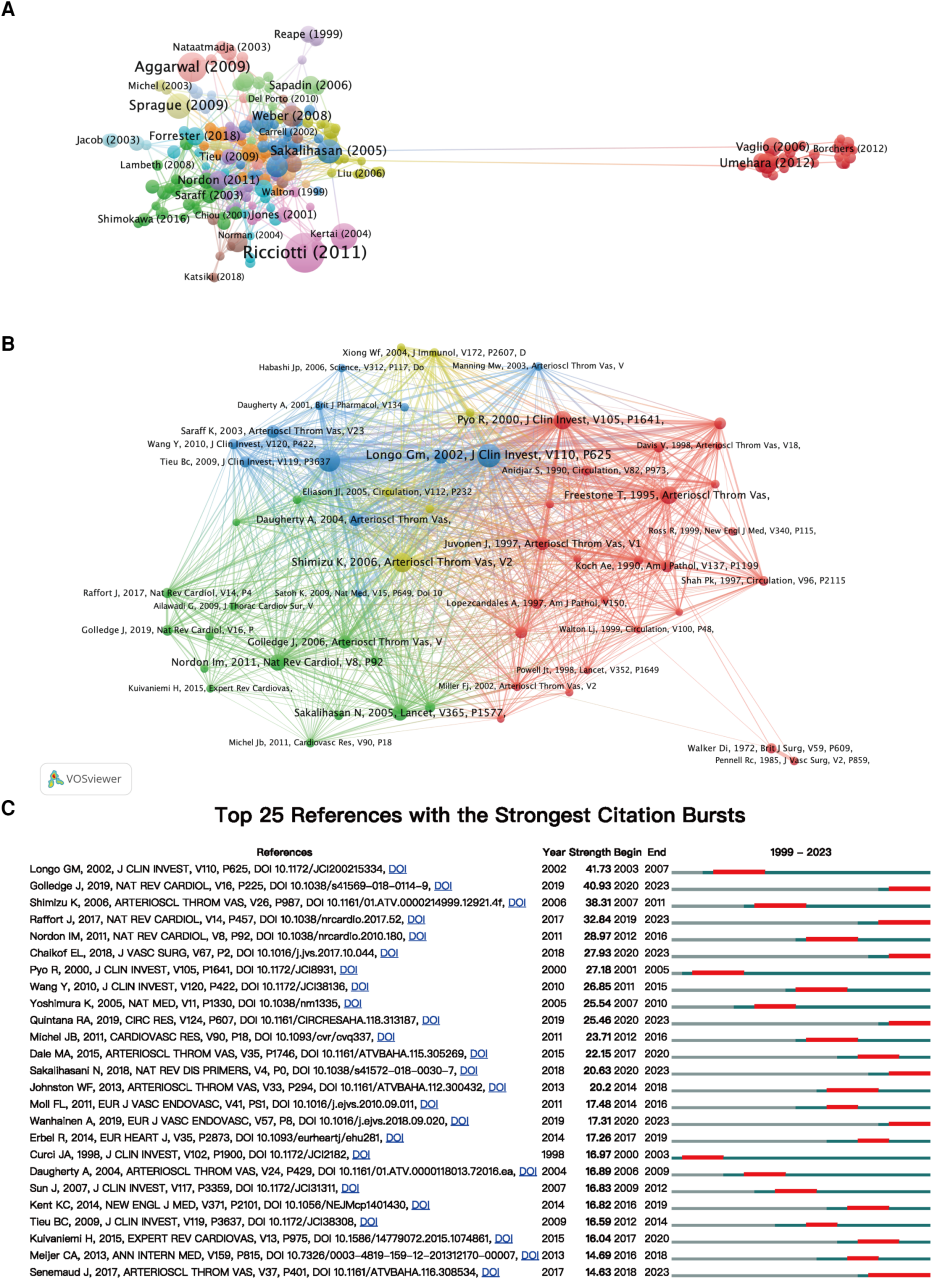
(**A**) Visualization analysis of documents with ≥100 citations. (**B**) Co-cited analysis of documents with ≥80 co-citations. (**C**) The top 25 references with the strongest bursts documents in this field.

In this research field, a total of 278 articles have been cited 100 times or more (Figure 7A). Further co-citation analysis was conducted using VOSviewer, visualizing influential articles (Figure 7B). Highly co-cited literature forms a crucial foundation in this investigative domain, providing substantial references and valuable insights. Among them, the top 10 most co-cited articles are listed in Table 6. The article titled “Matrix metalloproteinases 2 and 9 work in concert to produce aortic aneurysms” has been co-cited 391 times, “Angiotensin II promotes atherosclerotic lesions and aneurysms in apolipoprotein E-deficient mice” has been co-cited 348 times, “Inflammation and cellular immune responses in abdominal aortic aneurysms” has been co-cited 296 times, “Inflammatory cytokines in vascular dysfunction and vascular disease” has been co-cited 268 times, and “Inflammation and matrix metalloproteinases in the enlarging abdominal aortic aneurysm” has been co-cited 256 times.

**Table 6:** The top 10 documents with the most co-citations in this field.

Rank	Title	Journal	Publication year	IF	Co-citations
1	Matrix metalloproteinases 2 and 9 work in concert to produce aortic aneurysms	J Clin Invest	2002	15.9	391
2	Angiotensin II promotes atherosclerotic lesions and aneurysms in apolipoprotein E-deficient mice	J Clin Invest	2000	15.9	348
3	Inflammation and cellular immune responses in abdominal aortic aneurysms	Arterioscler Thromb Vasc Biol	2006	8.7	296
4	Targeted gene disruption of matrix metalloproteinase-9 (gelatinase B) suppresses development of experimental abdominal aortic aneurysms	J Clin Invest	2000	15.9	268
5	Inflammation and matrix metalloproteinases in the enlarging abdominal aortic aneurysm	Arterioscler Thromb Vasc Biol	1995	8.7	256
6	Pathophysiology and epidemiology of abdominal aortic aneurysms	Nat Rev Cardiol	2011	49.6	226
7	Abdominal aortic aneurysm	Lancet	2005	168.9	216
8	Elevated circulating levels of inflammatory cytokines in patients with abdominal aortic aneurysm	Arterioscler Thromb Vasc Biol	1997	8.7	176
9	Abdominal aortic aneurysm: pathogenesis and implications for management	Arterioscler Thromb Vasc Biol	2006	8.7	176
10	Mouse models of abdominal aortic aneurysms	Arterioscler Thromb Vasc Biol	2004	8.7	174

Additionally, we conducted burst citation analysis using CiteSpace to identify the reference articles that have captured researchers’ interest in the field of aortic aneurysm and inflammation within a certain period. The top 25 articles are displayed (Figure 7C). The paper titled “Matrix metalloproteinases 2 and 9 work in concert to produce aortic” ranked first (Strength = 41.73) and was published in The Journal of Clinical Investigation in 2002, with a citation burst from 2003 to 2007. The most highly cited paper in recent years (Strength = 40.93) is “Abdominal aortic aneurysm: update on pathogenesis and medical treatments” by Jonathan Golledge, published in the journal Nature Reviews Cardiology in 2019, with citation burst continuing to the present.

### 3.6. Analysis of keywords and evolution of hotspots

**Figure 8:**
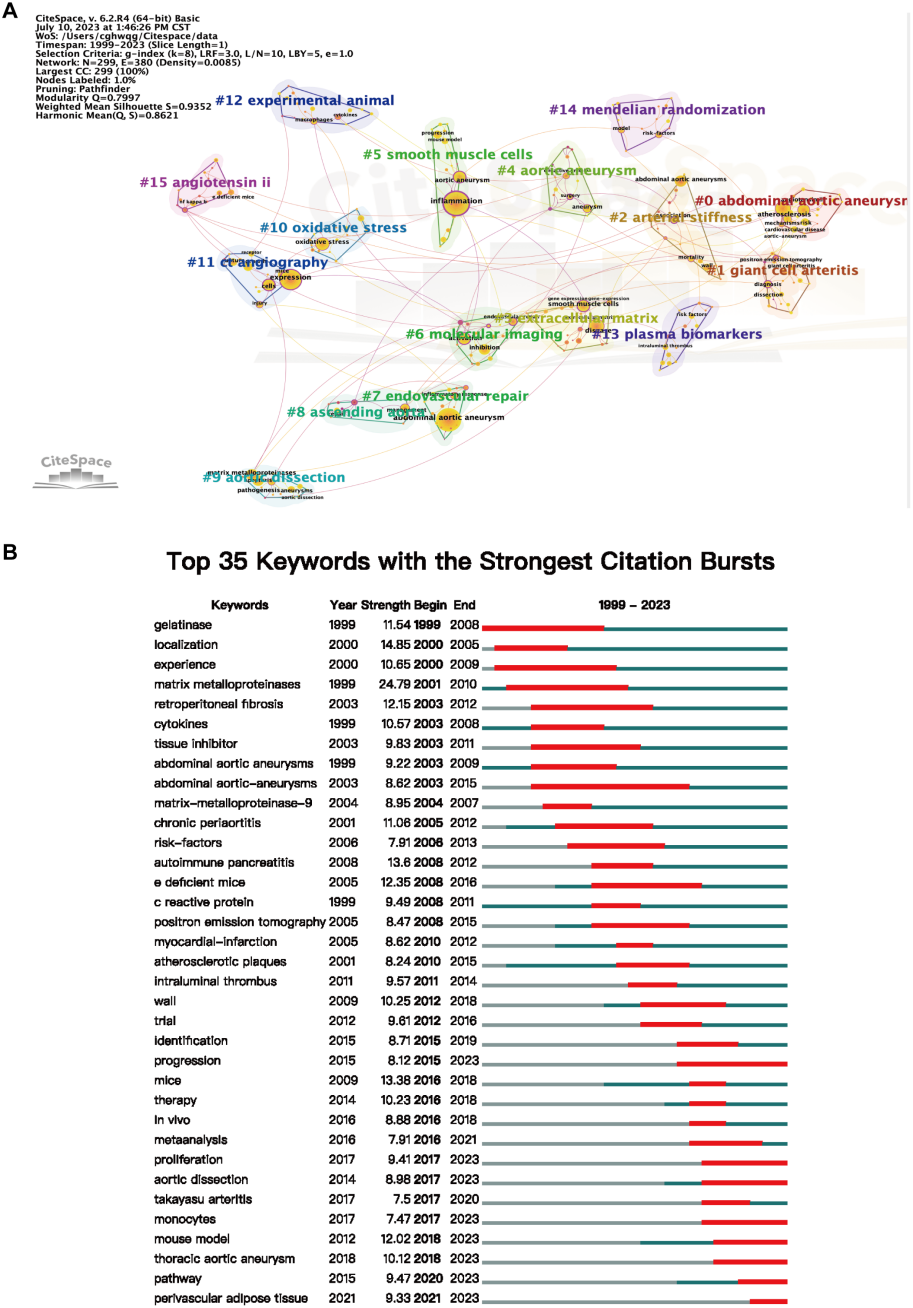
(**A**) visualization analysis of keyword clustering in the field of AA research related to inflammation, resulting in a total of 16 clustered groups. (**B**) Top 35 keywords with the strongest citation burst in this field.

A cluster analysis of all literature keywords in this study was conducted using the LSI algorithm based on CiteSpace keyword clustering analysis (Figure 8A). The keywords were divided into 16 clusters and named after the keyword with the highest frequency in each cluster. The smaller the ID number of the cluster, the larger the size of the cluster. Among them, “abdominal aortic aneurysm,” “giant cell arteritis,” “arterial stiffness,” and “smooth muscle cells” were among the largest clusters. Through CiteSpace, we performed keyword burst analysis on the top 35 keywords with the highest frequency (Figure 8B) to understand the frequency and duration of keyword appearances at different time points, thereby identifying the development trajectory of research hotspots. We found that “Monocytes,” “Progression,” and “Proliferation” were the most frequent and long-lasting research topics in the past five years.

**Figure 9:**
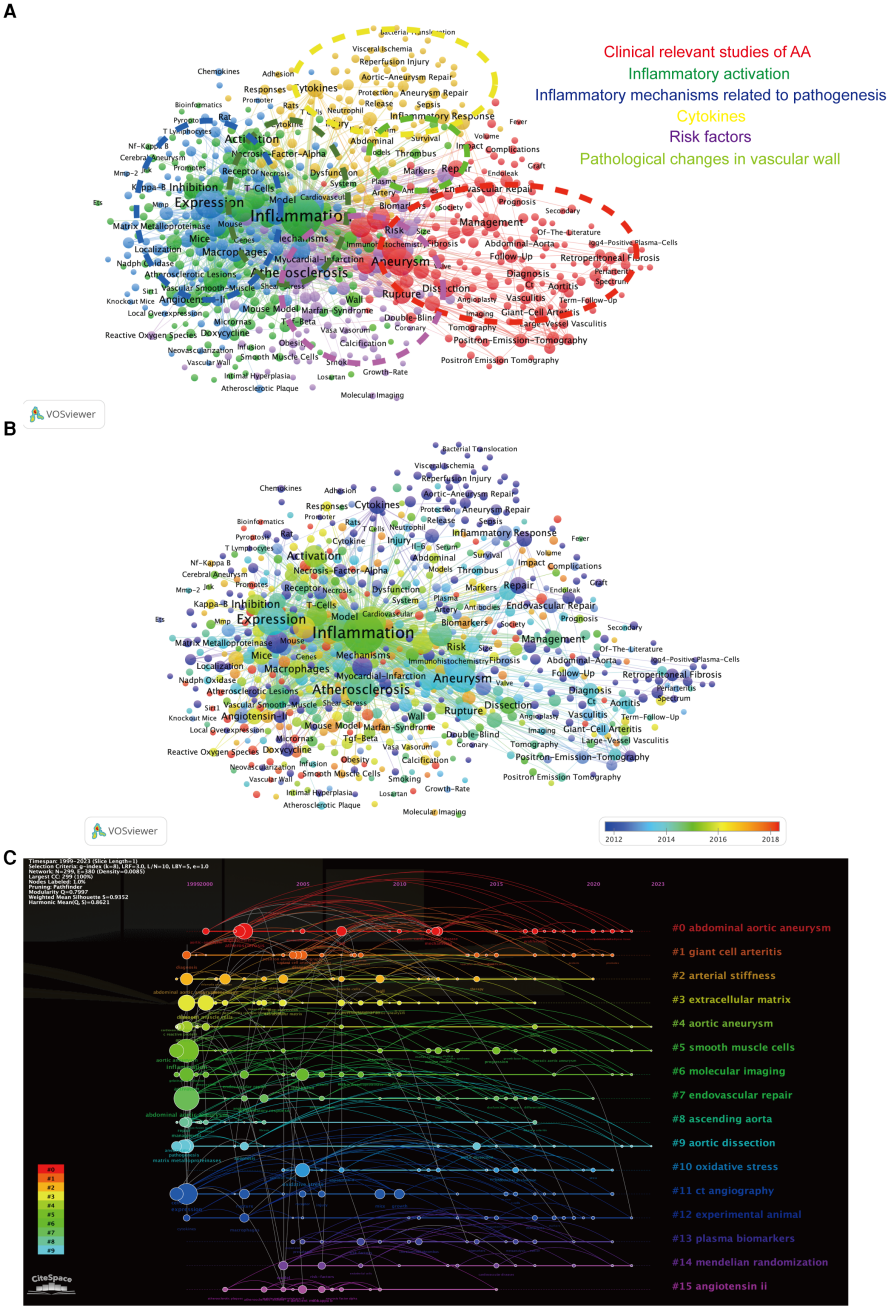
(**A**) Visualization analysis of keyword co-occurrence reveals six clustered groups based on keyword clustering. (**B**) Visualization of keywords at different time periods in keyword co-occurrence analysis. (**C**) The timeline graph of keyword clustering (LSI) was generated using CiteSpace.

Keyword co-occurrence analysis is a bibliometric analysis method used to explore the association and common occurrence of keywords in a research field. It helps researchers discover the correlation between keywords, reveal the connections between research topics, and identify the hotspots and trends in the research field. Using VOSviewer, we performed co-occurrence analysis on keywords (709 in total) that appeared at least 10 times in the titles and abstracts of the literature (Figure 9A). The analysis identified six major categories: “Clinical relevant studies of AA” (red), “Inflammatory activation” (green), “Inflammatory mechanisms related to pathogenesis” (dark blue), “Cytokines” (yellow), “Risk factors” (purple), and “Pathological changes in vascular wall” (cyan). In the “Inflammatory activation” cluster, “smooth muscle” and “oxidative stress” were core keywords, while in the “Inflammatory mechanisms related to pathogenesis” cluster, “macrophages” and “matrix metalloproteinases” were highly frequent core keywords. As shown in Figure 9B, the appearance time of the keywords was color-coded, and the label size represented the frequency of keyword occurrence. “Inflammatory activation” and “Inflammatory mechanisms related to pathogenesis” are hot topics that have emerged in the field of arterial aneurysm research in recent years. In the timeline graph of keywords, we found that “Smooth Muscle Cells”, “Oxidative Stress” and “Experimental Animal” are the most frequently mentioned keywords in recent years (Figure 9C).

## 4. Discussion

AA is a chronic arterial disease encompassing abdominal aortic aneurysm (AAA) and thoracic aortic aneurysm (TAA). Typically, this condition presents without obvious symptoms. However, as the aortic pathology progresses, it can lead to aortic rupture and aortic dissection, posing severe complications and life-threatening risks to patients. In some developed countries, approximately 1%–2% of males over 65 years old suffered from AAA (6, 7), and AAA accounts for 1.3% of deaths in men aged 65–85 (8), making it one of the common causes of mortality in the elderly (9). In contrast, although the prevalence of TAA is lower than that of AAA, patients with ruptured TAA have a significantly higher risk of mortality compared to AAA patients (10–13). Due to the inconspicuous clinical features of AA, early diagnosis poses challenges. Currently, there are no effective pharmacological treatments for AA, and surgical intervention remains the only viable approach. Presently, inflammatory responses are widely recognized as typical pathological changes in the development of AA. Numerous studies have unequivocally demonstrated a close association between aortic structural alterations in AA and inflammatory responses, with significant progress made in understanding the role of inflammation in AA (14, 15). However, it was not until recent years that researchers began to consider inflammatory responses as a key initiating factor in the AA pathological process (16, 17). Nonetheless, there remains a degree of uncertainty regarding the activating factors of immune cells within AA and the mechanisms underlying inflammatory responses. Therefore, it is crucial to analyze the current research status of AA in the context of inflammation and identify more meaningful research directions related to AA and inflammation.

To address the aforementioned issues and meet the research needs, this study employed the method of bibliometric analysis for the first time. Using tools such as CiteSpace, VOSviewer, Bibliometric, and other online analysis tools, the literature in this research field was analyzed. Visual and quantitative analysis of basic information such as the number of publications, countries of publication, research institutions and authors, journals, and keywords was conducted to summarize and summarize the research outcomes and advancements.

### 4.1. General overview of the research field

From January 1999 to April 2023, a total of 3,993 research papers were published in the field of AA and inflammation. The number of publications has steadily increased each year, indicating a growing recognition and attention to research in this field. Statistics on the number of publications per year by country reveal that the United States, China, Japan, the United Kingdom, and Germany were the major contributing countries in this field. Among them, the United States had the highest publication output, which has remained stable in recent years. China ranked second, but since 2013, the number of publications from China has rapidly increased, surpassing the United States and becoming the country with the highest annual publication output after 2020 (Figure 3A). This indicates that scholars in the United States have continued to delve into this field, while Chinese scholars have actively engaged in related research, driven by increased research funding and scholarly attention. Close collaborations exist among high-output countries, especially with frequent exchanges involving Chinese scholars. Institution analysis revealed that research institutions such as Harvard University, University of Kentucky, Stanford University, and Washington University in the United States were at the forefront in terms of publication output and citations, indicating their authoritative research funding and achievements. As a rising star in this field, Chinese research institutions such as Capital Medical University, Huazhong University of Science and Technology, and Chinese Academy of Medical Sciences emerged as the most influential and productive institutions in China, jointly leading the development of this field since 2016 with their counterparts in the United States (Figure 4). To gain a deeper understanding of the focal topics in this research field, researchers should explore the research directions and achievements of institutions such as Harvard University, University of Kentucky, Stanford University, and Washington University, while also paying attention to the current status and relevant literature of emerging research institutions like Capital Medical University, Huazhong University of Science and Technology, and Chinese Academy of Medical Sciences. Establishing collaborative relationships and jointly exploring the hot topics in this field are crucial. Author analysis revealed that Jonathan Golledge from James Cook University in Australia had the highest number of publications, followed by Alan Daugherty and Gilbert R. Upchurch Jr from the United States, and Guoping Shi from China. Jonathan Golledge, Alan Daugherty, and Guoping Shi were the top three authors with the highest co-citations, indicating their significant impact and substantial academic contributions in this field (Table 3). Further analysis of co-citation among authors showed that Jonathan Golledge and Alan Daugherty formed the most influential research group. Impact Factor (IF) and Journal Citation Reports (JCR) ranking are indicators used to evaluate the influence of journals, with high IF values and higher-ranking JCR zones representing journals with greater impact. In the research on AA and inflammation, 60% of the top 10 most influential journals belonged to the highest JCR zone (JCR1) and had high IF values, with the highest impact factor observed in the New England Journal of Medicine (Table 5). Furthermore, research related to AA and inflammation was mainly published in high-impact journals in the fields of immunology, molecular biology, basic medicine, and clinical medicine, such as Arteriosclerosis Thrombosis and Vascular Biology, Circulation, Journal of Clinical Investigation, and Journal of Vascular Surgery (Figure 6B). These visualized analysis data provide valuable references for researchers to identify research directions, track research hotspots, and guide future journal submissions.

### 4.2. Research trends and exploration of hot topics

Highly cited literature not only reflects its influence in the research field but also represents the cornerstone of a specific research area within a certain timeframe (18). In the early studies of this field, G. Matthew Longo discovered that the occurrence of AAA was significantly reduced in MMP-9 KO and MMP-2 KO mice using a CaCl_2_-induced AAA model. However, the occurrence of AAA in MMP-9 KO mice was significantly increased after reconstitution with wild-type macrophages, accompanied by inflammatory infiltration and aggravation of aortic pathology, suggesting the promotion of AAA development by MMP-9 secreted by macrophages (19). Additionally, in the early studies summarizing the role of inflammatory response in AAA, Koichi Shimizu pointed out, particularly in sporadic AA, the Th1-type immune response-related cytokines (such as IFN-γ) are crucial for inflammatory cell recruitment during early aortic pathology in AAA (20). In the continuous progression of AAA, Th2-type immune response-related cytokines and chemokines (such as IL-4) play an important role (21). It was suggested that early specific targeting of Th1-type cytokines, as well as inhibition of Th2-type immune response-related factors, could be potential therapeutic strategies for AAA progression.

In the past, the prevailing theory regarding the pathogenesis of AAA suggested that inflammation only played a significant role in about 5% of AAA cases (22). However, in 2019, Jonathan Golledge proposed a new theory on the pathogenesis of AAA in his paper titled “Abdominal aortic aneurysm: update on pathogenesis and medical treatments” published in the journal “Nature Reviews Cardiology”. In contrast to previous perspectives, Golledge emphasized the importance of disruption in the aortic immune environment mediated by genetic and environmental factors as key contributors to AAA development. He pointed out that innate immune cells such as neutrophils and monocyte/macrophages, as well as adaptive immune cells including T cells and B cells, may play crucial roles in the progression of AAA. Golledge also highlighted the potential of intervening in the innate or adaptive immune processes during AAA development as a promising direction for future therapeutic interventions (4). In his research, Golledge acknowledged that the efficacy of anti-inflammatory drugs in treating AAA has shown limited results in some clinical trials (4). This may be due to the involvement of multiple inflammatory pathways in AAA pathogenesis, making it challenging to achieve satisfactory outcomes with anti-inflammatory treatments targeting a single inflammatory cell or mechanism. Additionally, for older patients with AAA, broad anti-inflammatory treatments may carry other risks. Therefore, the development of safer and more gentle novel anti-inflammatory approaches, including neutralizing key pathogenic inflammatory factors, holds promise as a prospective avenue for research and development.

In bibliometric studies, researchers typically utilize methods such as keyword clustering and co-occurrence analysis to outline the knowledge network framework in the field. Visual analysis of keyword bursts and timeline graphs effectively assists researchers in quickly understanding research trends and identifying new research directions (18). Through VOSviewer's co-occurrence analysis of keywords (Figure 8A), we identified that “clinical studies of aneurysms,” “inflammation activation,” “inflammation-related pathogenesis,” “inflammatory factors,” “risk factors,” and “vascular wall pathological changes” are the major research topics in this field.

In the clustering cluster of “Clinical relevant studies of AA,” high-frequency keywords include “Abdominal Aortic Aneurysm,” “Management,” “Diagnosis,” “Follow-Up,” and “Therapy,” which have been consistently appearing since 2010. This indicates that inflammation has received attention in the research field of abdominal aortic aneurysm and has been extensively studied in clinical observations. Some clinical studies have found that certain cytokines and inflammation-related factors, such as IL-6 and VEGF-2, are associated with the occurrence, development, and related severe complications of AAA (23, 24). Early animal model studies discovered that doxycycline can effectively inhibit the progressive dilation of the aorta (25–27). Subsequently, researchers intervened with different doses of doxycycline in patients undergoing elective open AAA repair, and they found that doxycycline can effectively reduce the infiltration of neutrophils and cytotoxic T cells in the diseased aortic wall, decrease IL-4, IL-13, and colony-stimulating factor levels in aortic tissue. Short-term doxycycline treatment was suggested to inhibit the occurrence and development of AAA through specific anti-inflammatory effects (28). Additionally, a study found that in patients undergoing open AAA repair surgery, the combined use of HMG-CoA reductase inhibitors (such as simvastatin) and cholesterol absorption inhibitors (such as ezetimibe) can effectively reduce MMP-9 and IL-6 levels in the aortic wall (29). The current research status mentioned above indicates that researchers dedicated to studying AAA may have realized the involvement of inflammatory responses as a crucial factor in the occurrence and development of AAA. Investigating the role and mechanisms of inflammatory responses during the development of AAA may better explain the causes of AAA occurrence, development, and related complications. However, the current relevant clinical research in this field is still insufficient, particularly lacking large-sample clinical randomized controlled trials, which would provide more reliable clinical evidence. In the next stage, multicenter collaborations and large-sample clinical randomized controlled studies may help address the research challenges in this field.

In the co-occurrence analysis clustering of keywords “Inflammation activation” and “Inflammation-related pathogenic mechanisms,” several high-frequency keywords emerged, such as SMC, Endothelial-Cell, Macrophage, Monocytes, *T* cell, and Extracellular Matrix. These keywords showed a concentrated appearance after 2015, indicating a strong correlation. On one hand, this suggests that research focuses on these keywords as hot topics. On the other hand, it indicates that the development of AAA is the result of multicellular interactions, rather than the sole influence of individual cells or factors. Studies have proposed a novel communication network between aortic endothelial cells, macrophages, and SMCs in AAA. Aortic endothelial cells release ATP to macrophages and SMCs through Pannexin-1 channels, promoting intercellular communication and facilitating AAA development. In this process, activation of macrophage P2X7 receptors triggers the release of mtDNA, increased secretion of IL-1β and HMGB1, thereby exacerbating local inflammation in the aorta. Additionally, activation of SMC P2Y2 receptors and TRPV4 channels leads to SMC phenotype transformation (30). Research by Bo Liu's team found that certain components in the extracellular matrix, such as platelet response protein-1, promote AAA development by regulating the physiological activity of monocyte macrophages (31). Emerging single-cell sequencing studies in recent years have revealed the heterogeneity of SMCs, fibroblasts, endothelial cells, macrophages, and other immune cells in AAA development. Single-cell sequencing analysis of mouse AAA tissue by Bo Liu's team revealed a decrease in the proportion of SMCs and fibroblasts in AAA, while an increase in the proportion of macrophages, neutrophils, and dendritic cells, accompanied by characteristic transcriptional changes compared to cells in the normal aorta (32).

As research advances, increasing attention is being directed towards immune cells in the progression of abdominal aortic aneurysm (AA). Among these cells, neutrophils, one of the largest subsets of immune cells, are gaining prominence. Neutrophils exhibit rapid response capabilities, swiftly migrating to sites of damage or infection, thereby initiating immune reactions. Within the aortic lesion sites of AA, neutrophils constitute a major component of inflammatory cells. They contribute to arterial wall damage by releasing substances such as myeloperoxidase, matrix metalloproteinases, elastase, among others (33–35). Furthermore, activated neutrophils at these lesion sites can exacerbate the inflammatory response by forming extracellular traps, promoting the activation of Th17 cells and macrophages, leading to increased release of pro-inflammatory factors, and a reduction in vascular smooth muscle cells, thereby driving the progression of AA (36). Additionally, monocytes and macrophages, as the principal inflammatory cell populations in AA, play pivotal roles in the disease's progression. Monocytes are typically categorized into three subtypes based on CD14 and CD16 expression: classical monocytes (CD14^++^CD16^−^), intermediate monocytes (CD14^++^CD6^−^), and non-classical monocytes (CD14^+^CD16^++)^. Classical monocytes play a significant role in innate immunity and possess the potential to promote tissue repair (37, 38). CD14^++^CD16^−^ monocytes, on the other hand, have been found to exhibit phagocytic and pro-inflammatory characteristics (39, 40). Relevant research has shown a significant increase in CD14^++^CD16^−^ monocytes and a decrease in CD14^++^CD16^−^ monocytes in the peripheral blood of AA patients compared to healthy individuals. However, studies on the CD14^+^CD16^++^ monocytes have yielded inconsistent results (41, 42). The diversity of monocytes in AA indicates their relevance to the disease, and further investigation is warranted to comprehensively discuss the distinct functions of different monocyte subtypes in the pathogenesis of AA. Furthermore, with the continuous advancement of research techniques, there have been new insights into the origins of macrophages in tissue organs. Some macrophages in the heart and blood vessels originate from embryonic resident macrophages, which can self-renew through proliferation to maintain tissue homeostasis. Another portion of macrophages arises from the differentiation and residency of bone marrow-derived monocytes. These macrophages from different sources exhibit distinct physiological functions during the inflammatory response. Studies have indicated an increased number of macrophages with altered phenotypes in the full thickness of AA lesions, suggesting that macrophage increase and polarization play a critical role throughout the progression of AA (43). Both embryonic-origin and circulation-derived macrophages participate in macrophage infiltration and phenotypic changes in the diseased aorta (44). However, the role of macrophages from different sources, especially embryonic-origin macrophages, in the progression of AA has made some progress but still requires further research to elucidate their roles and pathogenic mechanisms in AA.

Based on the results of the keyword visualization analysis, it suggests that researchers may need to approach the study of AAA from multiple perspectives to better understand its dynamic process of occurrence and development. In the literature of this research field, “Cytokines” is one of the main keyword clusters in co-occurrence analysis. Additionally, in the timeline visualization of keywords, “plasma biomarkers” is a keyword cluster that has attracted researchers’ attention in the past 15 years. Increasing clinical and basic research has confirmed the relevance of inflammatory-related cytokines as important factors involved in the development of AA. For example, TNF can amplify immune response and inflammatory damage, leading to SMC injury or even death, promoting increased release of MMPs, and disrupting the extracellular matrix, playing a crucial role in the process of aortic dilation (45). Clinical studies have indicated that elevated levels of TNF in the serum of AAA patients are significantly associated with AAA symptoms, and levels of IL-6 and IL-8 are positively correlated with aneurysm diameter (46). Increased levels of IFN-γ in the serum of AAA patients are positively correlated with the rate of aneurysm expansion (47). IFN-γ is a signature cytokine of Th1 cells that can inhibit Th2 immune response. However, some studies have shown increased expression of Th2 cell inflammatory cytokines, such as IL-4, IL-5, and IL-10, in the diseased arterial tissues of AAA patients (48). In some AAA patients, plasma levels of IFN-γ, IL-17A, and C-reactive protein are positively correlated with the cross-sectional area of AAA, while IL-10 is positively correlated with the growth rate of the aneurysm, and increased CRP levels indicate an increased risk of death in AAA patients (49). Inflammatory response plays a significant role in the formation of AAA, and cytokines generated during the inflammatory response are gradually being recognized as biomarkers for assessing disease progression and risk levels (50–53). However, the exact role and mechanisms of inflammatory-related cytokines in AA disease have not been fully elucidated. Therefore, targeted intervention against inflammatory cytokines associated with AA holds great promise as a preventive and therapeutic approach for disease development and warrants further discussion.

We conducted a keyword burst analysis and found that “Monocytes,” “Progression,” and “Proliferation” have been frequent keywords in recent years. The inflammatory mechanisms of monocytes/macrophages have received significant attention in AA disease research. In an ANGII-induced mouse model of hypertension, inhibiting the polarization of M2 macrophages in the aorta reduced abnormal collagen deposition and alleviated elastin fiber fragmentation, effectively improving aortic vascular remodeling (54). M1 macrophages also play an important role in the development of AA. During AAA formation, damage to elastic fibers in the extracellular matrix leads to increased release of elastin-derived peptides, recruitment of monocytes/macrophages, and induction of their transformation into M1 phenotype, promoting AAA progression (55). Studies have also found that in a mouse model of CaCl_2_-induced AAA, TNF-α plays a more significant role than IL-1β in polarizing M1 macrophages during disease progression, and blocking TNF-α activation in monocytes/macrophages can more effectively inhibit AAA development (56). Different phenotypes of macrophages (M1, M2) play important roles in different stages of AA development and in different regions of the diseased artery (57, 58). There is evidence to suggest that modulating macrophage polarization through targeted drug interventions or cell therapy effectively inhibits AA progression by maintaining their homeostasis (59–61). Therefore, maintaining the homeostasis of local macrophages may be an effective approach to restrain AA progression. T lymphocytes in adaptive immune cells play important roles in regulating the immune environment and promoting tissue repair in autoimmune and other inflammation-related diseases. Currently, there is some evidence suggesting that T cells also play a significant role in the development of AA, but more clinical and basic research is needed to explain their functions and effects in AA. Further in-depth research on the role and mechanisms of T cells in AA may provide more insights for interventions in AA.

SMCs constitute a major cellular component of the arterial wall and are primarily located in the middle layer of the aortic wall. With the progression of research, the heterogeneity of aortic SMCs has been gradually revealed. Aortic SMCs mainly originate from the secondary heart fields (SHF), cardiac neural crest (CNC), as well as somitic and splanchnic mesoderm layers (62). SMCs in the aortic root mainly arise from SHF, while those in the arch originate primarily from CNC (63). SMCs in the descending aorta below the arch are derived from the mesoderm (64). Recently, studies by Professor Alan Daugherty's team have indicated that SHF-derived SMCs play a crucial role in maintaining aortic vascular stability through the low-density lipoprotein receptor-related protein 1 (LRP1) and transforming growth factor-β (TGF-β) signaling pathways (65). Research in ANGII-induced AA models, using TGF-β antibody 2G7 to block TGF-β and tamoxifen injection into Acta2-CreERT^2+/0^ Tgfbr2^flox/flox^ mice, revealed that TGF-β exerts protective effects in the abdominal and thoracic aorta through distinct pathways (66). Further scRNA-seq analysis showed that Malat1^+^ SMCs are mainly distributed in the abdominal aorta, where they play a significant role in AAA. Knocking out the Malat1 gene in SMCs significantly inhibited ANGII-induced AAA formation, reducing aortic inflammation and MMP production in SMCs (67). Supplementary scRNA-seq results provided additional insights into the heterogeneity of aortic SMCs. Unlike traditional views, current research suggests significant intercellular differences among different subtypes of aortic SMCs, primarily related to genes associated with contraction, inflammation, migration, and proliferation (67–69). Clinical data indicate that the incidence of TAA is lower than that of AAA (10, 12, 13).

Combining these studies, we hypothesize that the different origins of aortic SMCs may lead to differences in the mechanisms and incidence rates between TAA and AAA. However, there is currently a lack of reliable research results to support this hypothesis, making it an intriguing research direction. Consequently, our current theoretical research revolves around this hypothesis. Although scRNA-seq technology has identified multiple subgroups of aortic SMCs, there is still a lack of related studies conducting in-depth analysis on SMCs from different parts of the aorta to clarify whether the differentiation of aortic SMCs in normal and pathological states is influenced by their origin and whether the mechanisms related to SMCs and inflammation are affected by the source of SMCs.

Additionally, our keyword clustering and burst analysis using CiteSpace indicate that keywords related to TAA, such as “Ascending Aorta,” and “Thoracic Aortic Aneurysm,” are highlighted. However, compared to keywords related to AAA, the frequency of TAA-related keywords is significantly lower (see Supplementary Table S1). This suggests a relatively low level of attention to TAA in this field, with research primarily focused on AAA. Furthermore, existing animal models for aortic aneurysms predominantly focus on AAA (70, 71), which may lead to some biases in the understanding of the mechanisms of TAA and AAA. Important reviews on AA and inflammation by Jonathan Golledge and Jean Senemaud respectively summarize common animal models involved in this field (4, 72). Currently, common animal models for TAA include BAPN, ANGII, and genetic TAA mice (FBN1^C1039G+/−^), while common animal models for AAA are induced through high-fat diet, ANGII, CaCl_2_, or elastase. However, these animal models cannot perfectly mimic the pathological processes of human TAA/AAA diseases. Therefore, improving animal models for TAA and AAA is crucial for further studying the pathogenic mechanisms of TAA and AAA.

### 4.3. Advantages and shortcomings

We conducted a systematic analysis of research in the field of AA and inflammation-related studies using bibliometric methods for the first time. In the analysis process, we employed various mainstream tools, including CiteSpace, VOSviewer, R package “bibliometric,” and online platforms, to conduct an in-depth analysis of the literature. The research results are stable and reliable. Through this bibliometric study, we are able to provide a more comprehensive and objective perspective than traditional review articles. This will contribute to a better and faster understanding of the research frontier by ourselves and other researchers in the field, collectively filling the gaps in research. However, due to the limitations of bibliometric tools, paper types, and language, we may overlook valuable newly published research findings and miss out on meaningful research papers published in languages other than English.

## 5. Conclusion

We conducted the first-ever bibliometric analysis of global research on AA and inflammation since 1999. We analyzed the publication trends, regional distribution, and collaboration among institutions in this research field. Additionally, we identified influential journals and valuable research findings, selecting interesting perspectives for discussion. Currently, there is an increasing focus on the relationship between AA and inflammatory responses. However, existing studies primarily focus on the impact of individual factors or single cells on AA, and the incomplete disease models may limit the comprehensive understanding of AA pathogenesis. In future research, researchers can strengthen national and institutional collaboration to facilitate multicenter cooperation and increase the clinical sample size for clinical trials. Furthermore, it is important to pay greater attention to the interactions between immune cells (including macrophages, T cells, and B cells) and endothelial cells, fibroblasts, and smooth muscle cells, as well as further refine the disease models for AA. This will provide more meaningful insights for exploring new therapeutic targets for AA.

## Data Availability

The raw data supporting the conclusions of this article will be made available by the authors, without undue reservation.
