# Supplementary material for: Global research status analysis of the association between aortic aneurysm and inflammation: a bibliometric analysis from 1999 to 2023
Source: Front Cardiovasc Med. 2023 Dec 4;10:1260935. doi: 10.3389/fcvm.2023.1260935 (PMC10725951; doi:10.3389/fcvm.2023.1260935)
Supplement: Supplementary file 1 [file Table1.docx]

Supplementary Material

Global Research Status Analysis of the Association Between Aortic Aneurysm and Inflammation: A Bibliometric Analysis from 1999 to 2023

Qiuguo Wang*, Guihuan Chen, Zhen Qi, Yifan Zeng

*** Correspondence:**

Hao Tang, [dr.tanghao@csu.edu.cn](mailto:dr.tanghao@csu.edu.cn)

Ling Tan, [dr.tanling@csu.edu.cn](mailto:dr.tanling@csu.edu.cn)

# Supplementary Data

As of September 30, 2023, we conducted a literature search in the Science Citation Index Expanded (SCI-E) of the Web of Science Core Collection (WoSCC) database. Studies related to inflammatory response in AAA were retrieved by TS= (AAA OR abdominal aortic aneurysm) AND TS= (inflammatory OR inflammation OR inflammations). By searching TS= (TAA OR thoracic aortic aneurysm) AND TS= (inflammatory OR inflammation OR inflammations), literature related to the association between TAA and inflammatory response was collected. We only included literature data that met the criteria of being original articles and reviews, written in English. In total, 1,101 TAA-related and 2,819 AAA-related literature articles were included. The literature full records, along with their cited references, were exported and organized in plain text format. The organized literature data was subjected to visual analysis using tools such as CiteSpace, VOSviewer.

A cluster analysis of all literature keywords in this study was conducted using the LSI algorithm based on CiteSpace keyword clustering analysis. The keywords from the TAA-related literature were categorized into 13 clusters and named after the keywords with the highest frequency in each cluster (Figure 1A). The smaller the ID number of the cluster, the larger the size of the cluster. Among them, "aneurysm repair," "marfan syndrome," "thioacetamide," and "hepatic stellate cells" were among the largest clusters. Through CiteSpace, we performed keyword burst analysis on the top 25 keywords with the highest frequency (Figure 1B) to understand the frequency and duration of keyword appearances at different time points, thereby identifying the development trajectory of research hotspots. We found that "macrophages," "extracellular matrix," and " hepatic encephalopathy" were the most frequent and long-lasting research topics in the past five years.

The keywords from the AAA-related literature were categorized into 16 clusters (Figure 1C). Among them, "abdominal aortic aneurysm", "intraluminal thrombus", "inflammatory response" and "nicotinic acid" were among the largest clusters. Through CiteSpace, we performed keyword burst analysis on the top 25 keywords with the highest frequency (Figure 1D). "Mouse model", "monocytes" and " endothelial dysfunction " have been hot keywords in recent years.

Using VOSviewer, we conducted co-occurrence analysis of keywords appearing 10 times or more in TAA-related literature and keywords appearing 20 times or more in AAA-related literature. This resulted in the formation of 4(Figure 2A) and 5(Figure 2B) keyword clusters respectively. Further extraction was done on meaningful and high-frequency keywords within the formed keyword cluster results (Table 1 and 2). We found substantial overlap in the keyword hotspots between TAA and AAA. This indicates a high degree of similarity in previous research between these two conditions.

# Supplementary Figures and Tables

## Supplementary Figures

Supplementary Figure 1. (A) Visualization analysis of keyword clustering in the field of TAA research related to inflammation, resulting in a total of 13 clustered groups. (B) Top 25 keywords with the strongest citation burst in the field of TAA research related to inflammation. (C) Visualization analysis of keyword clustering in the field of AAA research related to inflammation, resulting in a total of 16 clustered groups, (D) and the top 25 keywords with the strongest citation burst in this field.

Supplementary Figure 2. (A) Visualization analysis of keyword co-occurrence in TAA-related literature reveals four clustered groups based on keyword clustering. (B) Visualization analysis of keyword co-occurrence in AAA-related literature reveals four clustered groups based on keyword clustering.

## Supplementary Table

Table 1: High-frequency keywords in TAA-related literature

| label | cluster | Total link strength | Occurrences |
| --- | --- | --- | --- |
| Aneurysm | 1 | 491 | 119 |
| Dissection | 1 | 404 | 76 |
| Atherosclerosis | 1 | 251 | 50 |
| Oxidative Stress | 2 | 982 | 160 |
| Thioacetamide | 2 | 1054 | 158 |
| Activation | 2 | 691 | 112 |
| Apoptosis | 2 | 533 | 82 |
| Pathogenesis | 3 | 306 | 50 |
| Smooth-Muscle-Cells | 3 | 273 | 50 |
| Matrix Metalloproteinases | 3 | 273 | 45 |
| Marfan-Syndrome | 3 | 190 | 36 |
| Inflammation | 4 | 1821 | 348 |
| Liver Fibrosis | 4 | 638 | 100 |
| Hepatic Stellate Cells | 4 | 333 | 54 |
| Macrophages | 4 | 184 | 31 |

Table 2: High-frequency keywords in AAA-related literature

| label | cluster | Total link strength | Occurrences |
| --- | --- | --- | --- |
| Smooth-Muscle-Cells | 1 | 1808 | 293 |
| Rupture | 1 | 902 | 147 |
| Risk-Factors | 1 | 760 | 130 |
| Repair | 1 | 454 | 110 |
| Matrix Metalloproteinases | 2 | 1610 | 257 |
| Pathogenesis | 2 | 1506 | 249 |
| Oxidative Stress | 2 | 1467 | 240 |
| Macrophages | 2 | 991 | 153 |
| Inflammation | 3 | 6049 | 1049 |
| Growth | 3 | 902 | 132 |
| Endovascular Repair | 3 | 390 | 94 |
| Atherosclerosis | 4 | 2959 | 479 |
| Cytokines | 4 | 783 | 127 |
| Abdominal Aortic-Aneurysm | 5 | 1460 | 340 |
| Angiotensin II | 5 | 672 | 100 |
