# Supplementary figures and images for: Global research status analysis of the association between aortic aneurysm and inflammation: a bibliometric analysis from 1999 to 2023
Source: Front Cardiovasc Med. 2023 Dec 4;10:1260935. doi: 10.3389/fcvm.2023.1260935 (PMC10725951; doi:10.3389/fcvm.2023.1260935)

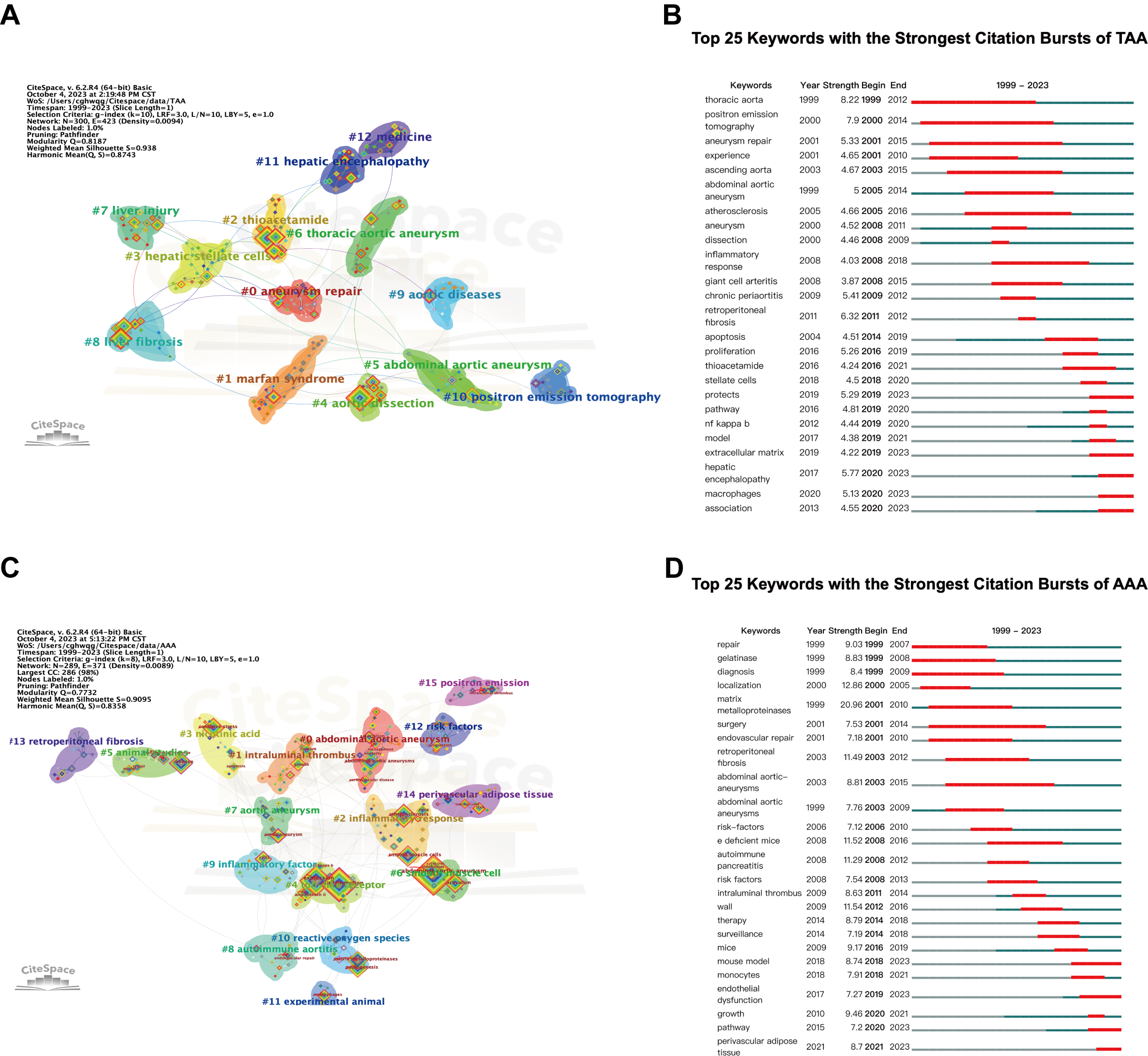

Supplement: Supplementary file 3 [file Image1.tif]

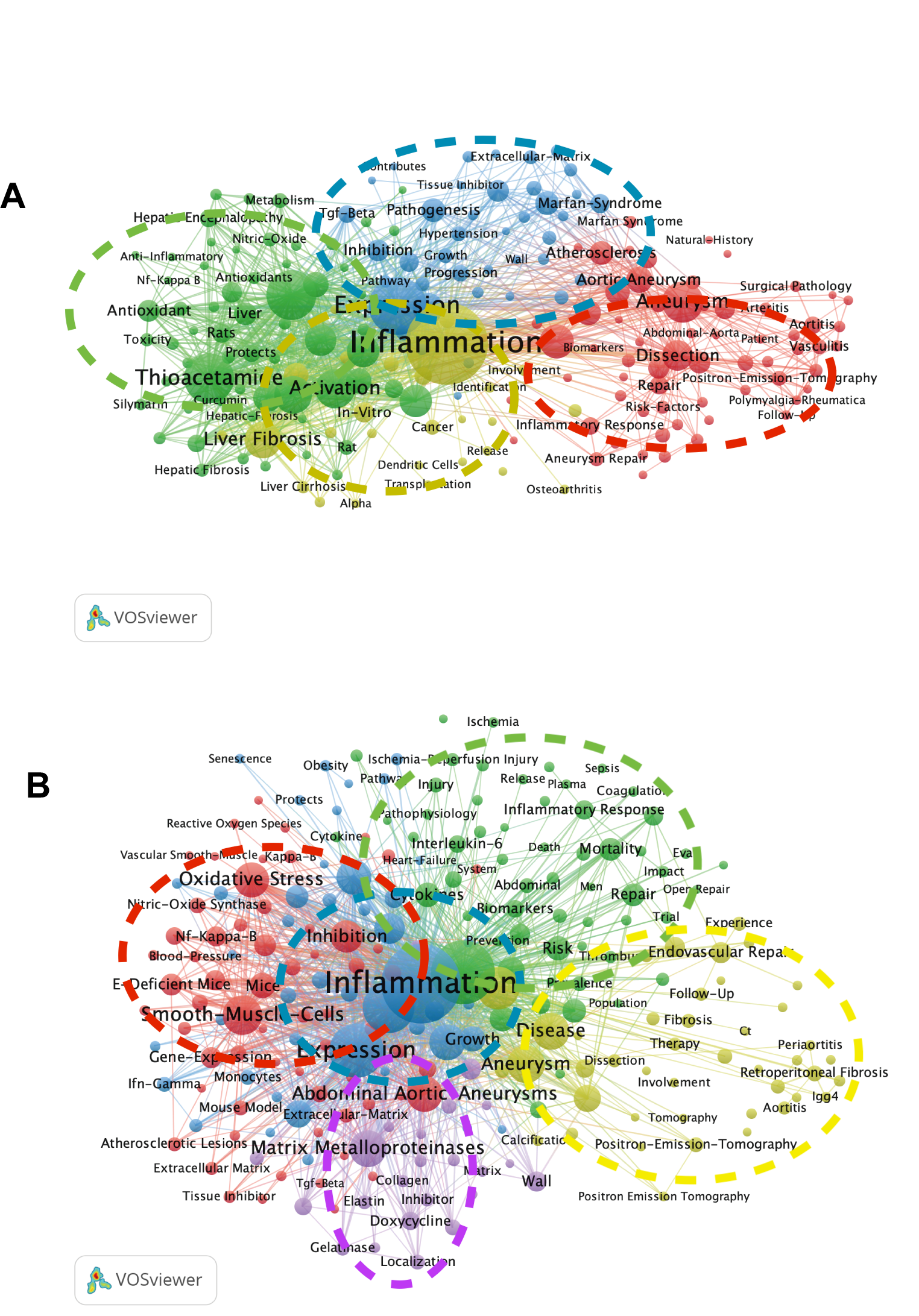

Supplement: Supplementary file 4 [file Image2.tif]
